# The trafficking pathway of a wheat storage protein in transgenic rice endosperm

**DOI:** 10.1093/aob/mcu008

**Published:** 2014-03-05

**Authors:** Maria Oszvald, Laszlo Tamas, Peter R. Shewry, Paola Tosi

**Affiliations:** 1 Department of Plant Physiology and Molecular Plant Biology, Eötvös Loránd University, Budapest, Hungary; 2 Plant Biology and Crop Science, Rothamsted Research, Harpenden, UK; 3 School of Agriculture, Policy and Development, University of Reading, UK

**Keywords:** HMW-GS, immunolocalization, protein trafficking, protein bodies, cereal endosperm, transgenic rice, *Oryza sativa*, wheat, *Triticum aestivum*

## Abstract

**Background and Aims:**

The trafficking of proteins in the endoplasmic reticulum (ER) of plant cells is a topic of considerable interest since this organelle serves as an entry point for proteins destined for other organelles, as well as for the ER itself. In the current work, transgenic rice was used to study the pattern and pathway of deposition of the wheat high molecular weight (HMW) glutenin sub-unit (GS) 1Dx5 within the rice endosperm using specific antibodies to determine whether it is deposited in the same or different protein bodies from the rice storage proteins, and whether it is located in the same or separate phases within these.

**Methods:**

The protein distribution and the expression pattern of HMW sub-unit 1Dx5 in transgenic rice endosperm at different stages of development were determined using light and electron microscopy after labelling with antibodies.

**Key results:**

The use of HMW-GS-specific antibodies showed that sub-unit 1Dx5 was expressed mainly in the sub-aleurone cells of the endosperm and that it was deposited in both types of protein body present in the rice endosperm: derived from the ER and containing prolamins, and derived from the vacuole and containing glutelins. In addition, new types of protein bodies were also formed within the endosperm cells.

**Conclusions:**

The results suggest that the HMW 1Dx5 protein could be trafficked by either the ER or vacuolar pathway, possibly depending on the stage of development, and that its accumulation in the rice endosperm could compromise the structural integrity of protein bodies and their segregation into two distinct populations in the mature endosperm.

## INTRODUCTION

Wheat (*Triticum* sp.) is the most important crop in Europe, being used for both food processing and livestock feed. Its success is due partly to its adaptability, giving high yields over a range of environments. However, its success as a food crop is also due to the unique properties of the grain storage proteins. These form the gluten fraction which confers the unique viscoelastic properties which allow wheat dough to be processed to form bread, noodles, pasta and many other processed foods. The well-established systems for wheat grain production, storage and fractionation also make it attractive for the production of novel components, such as high value pharmaceutical products or raw materials for industry. In order to exploit all of these possibilities, it is necessary to understand the pathways and mechanisms which determine the synthesis, processing, trafficking and deposition of storage components in the developing grain. This has been facilitated in wheat by the development of efficient transformation systems for bread wheat and the identification of strong endosperm-specific promoters (Shewry and Jones, 2005; Wiley *et al.*, 2007).

The trafficking of proteins in the endoplasmic reticulum (ER) is a topic of considerable interest since this organelle serves as an entry point for proteins destined for other organelles, as well as for the ER itself. A unique feature of plants is that they are able to store proteins in the ER in addition to other endomembrane compartments, and the deposition of such storage proteins provides important sources for both human and animal nutrition.

The storage proteins of wheat (also known as wheat prolamins) are classically divided into two groups, the monomeric gliadins and polymeric glutenins (Shewry and Halford, 2002), with the latter being of particular interest as they are the major determinants of dough strength (elasticity) which is the most important quality parameter for breadmaking. The glutenin polymers consist of two types of sub-unit, called low molecular weight (LMW) and high molecular weight (HMW) glutenin sub-units. The proportion of glutenin polymers with high molecular mass is related to dough strength and this is determined, at least in part, by allelic variation in the composition of the HMW sub-units (Payne, 1987; Shewry *et al.*, 2003). However, environmental effects on dough strength also occur, which may be due to impacts on the assembly of the polymers in the developing grain (Ma *et al.*, 2005; Wan *et al.*, 2008).

The gluten proteins of wheat are synthesized on the rough ER and co-translationally inserted into the lumen. They subsequently become deposited in protein bodies, but the origin of these bodies has long been the subject of debate. Electron microscopy (EM) has provided convincing evidence for the presence of gluten proteins inside vesicles associated with the Golgi apparatus, suggesting that storage proteins may pass through the Golgi in their transport from the ER to the vacuoles (Parker and Hawes, 1982; Kim *et al.*, 1988; Loussert *et al.*, 2008) where they form protein deposits. However, some studies also showed protein deposits in small bodies surrounded by the ER membrane (Parker, 1980; Campbell *et al.*, 1981; Tosi *et al.*, 2009), indicating that aggregation of storage proteins into protein bodies may also occur within the ER, as demonstrated for prolamins of maize and rice (Krishnan *et al.*, 1986; Shigemitsu *et al.*, 2013) and more recently suggested by expression of wheat storage proteins in tobacco cells (Francin-Alami *et al.*, 2011, 2013). These conflicting results may be reconciled by the existence of two different types of protein body with different densities, called light protein bodies and dense protein bodies, which accumulate simultaneously and independently in wheat endosperm cells (Rubin *et al.*, 1992), and it is now widely accepted that two routes of protein body formation (translocation via the ER and Golgi to the vacuole and direct accumulation in the ER) may operate in the wheat endosperm. It has also been suggested that the two routes may differ in their importance during grain development, with accumulation within the ER becoming more prominent at later stages, and individual proteins following one or both routes (Tosi *et al.*, 2009; Tosi, 2012). Therefore, while all gluten proteins are synthesized on polyribosomes attached to the ER, their subsequent transport, deposition and accumulation into protein bodies may depend on the properties of the proteins themselves (particularly their insolubility in aqueous solvents) and on the developmental stage of the endosperm. It is probable that the folding and assembly of the gluten proteins is assisted by ER lumenal proteins such as the enzyme protein disulfide isomerase (PDI) and the molecular chaperone-binding protein (BiP), although this is still not conclusively established (Grimwade *et al.*, 1996; DuPont *et al.*, 1998).

Whereas prolamins are the major storage proteins in wheat, rice (*Oryza sativa*) preferentially accumulates glutelin proteins, which are related to the 11S globulin storage proteins of dicotyledonous plants. In rice, 60 − 80 % of total seed protein is composed of glutelins and 20 − 30 % of prolamins. Rice glutelins are synthesized as precursor polypeptides, which are post-translationally cleaved into two smaller sub-units. The two sub-units of glutelins are classified as acidic (a) or basic (b) sub-units with apparent molecular weights of 30–39 and 19–25 kDa, respectively. The glutelin subunits are able to form large, insoluble macromolecular complexes stabilized by hydrophobic interaction, hydrogen bonding and disulfide bonds between the polypeptides in the presence of PDI (Takemoto *et al.*, 2002; Van der Borght *et al.*, 2006).

Rice prolamins differ from the prolamins of most other cereals, in terms of both molecular weight and amino acid sequence (Muench *et al.*, 1999; Shewry and Casey, 1999). According to their mobility on polyacrylamide gels, they are classified into three groups: the 10, 13 and 16 kDa groups, with the 13 kDa prolamins further classified into three sub-groups (classes I, II and III) on the basis of their cysteine residue content.

Rice prolamins and glutelins are deposited into distinct protein bodies of different origin (Muench and Okita, 1997), and several studies have indicated a close relationship between the ER site of RNA translation and the final site of protein deposition and the importance of RNA targeting to specific ER sub-domains for the efficient transport to the vacuole or packaging into ER-derived protein bodies (Li *et al.*, 1993; Choi *et al.*, 2000; Saito *et al.*, 2009; Washida *et al.*, 2012). The prolamins (which are related to the wheat gluten proteins) aggregate inside the ER lumen aided by the chaperone BiP and form type-I protein bodies (PB-Is) (Li *et al.*, 1993) while the glutelins are translated on separate sub-domains of the ER and transported via the Golgi apparatus and vesicles into type-II protein bodies (PB-IIs) of vacuolar origin. Post-translational processing of the glutelin precursor protein into mature acidic and basic polypeptides occurs in PB-IIs by the action of a specific asparaginyl endopeptidase (Muntz, 1998). This clear segregation of the rice storage proteins into two populations of protein body means that transgenic expression in rice can be used to determine whether the wheat storage proteins contain targeting trafficking signals that are recognized by the cell secretory system.

We have therefore developed transgenic rice lines expressing the wheat HMW sub-unit 1Dx5 protein (Oszvald *et al.*, 2007), which is present in PBs of both vacuolar and ER origin in wheat (Levanony *et al.*, 1992), although concentrated in the latter, and used specific antibodies to demonstrate its distribution between and within tissues and protein bodies in the developing rice grain.

## MATERIALS AND METHODS

### Plant materials

The production of transgenic rice (*Oryza sativa*) lines expressing HMW sub-unit 1Dx5 of wheat (*Triticum aestivum*) under the control of its own starchy endosperm-specific promoter has been described previously (Oszvald *et al.*, 2007). Seeds were harvested at 7, 12, 14, 17, 20 and 25 days after flowering (daf).

### Antibodies

Antibodies to purified rice glutelins and prolamins (Krishnan and Okita, 1986) were kindly provided by Professor Okita. For the detection of the transgenic HMW-GS in rice, two different antibodies were used: the monoclonal antibody IFRN 1602 (Mills *at al*., 2000), specific for x-type HMW-GSs, and the polyclonal anti-R2-HMW (Denery-Papini *et al.*, 1996), recognizing a specific epitope conserved in all HMW-GSs.

### Extraction of seed protein and detection of HMW Dx5

Freeze-dried seeds were ground into fine powder. Total proteins were extracted by vortexing for 15 min with 500 µL of extraction buffer [1 m Tris–HCl (pH 8·0) containing 0·4 (w/v) iodoacetamide and 1 % (w/v) dithiothreitol (DTT)], followed by centrifugation at 10 000 *g* for 20 min. Rice storage proteins were extracted sequentially from mature seeds as described by Yang *et al.* (2007). Briefly, the stepwise protein extraction was carried out by the removal of albumins and globulins with 500 µL of saline buffer (0·5 m NaCl, 10 mm Tris–HCl, pH 7·5) followed by removal of cysteine-poor prolamins with 500 µL of 60 % (v/v) *n*-propanol solution and cysteine-rich prolamins with the same solution but containing 1 % (w/v) DTT. Each extraction step was accomplished by re-suspending seed powder in the solution. Finally the proteins were extracted from the residues with 500 µL of urea-SDS buffer (50 mM Tris–HCl, pH 6·8, 8 m urea, 4 % SDS, 5 % 2-mercaptoethanol, 20 % glycerol). Proteins were separated by SDS–PAGE according to Laemmli (1970). HMW sub-unit 1Dx5 was detected using rabbit anti-HMW-GS antibodies in combination with horseradish-conjugated peroxidase-labelled anti-rabbit IgG antibody (Sigma A-3687).

### Preparation of specimens for microscopy

Transverse sections of developing rice grain (1 mm thick) were cut with a clean double-sided razor blade, using a sliding motion to avoid crushing the seed, and fixed for 48 h in 4 % (v/v) paraformaldehyde and 2·5 % (v/v) glutaraldehyde in phosphate-buffered saline at pH 7·2. The fixed samples were dehydrated, embedded in LR White resin (medium grade acryl resin; London Resin, Berkshire, UK), and then sectioned.

Sections for microscopy were prepared using a Reichert–Jung Ultracut microtome. For light and fluorescence microscopy, approx. 1 µm thick sections were cut and collected on polylysine-coated multiwell slides. For electron microscopy, ultrathin sections were collected on Formvar-coated mesh nickel grids, stained with uranyl acetate [saturated solution in 2 % (v/v) distilled water] for 2 min, washed three times and examined with a JEOL JEM2010 transmission electron microscope.

### Immunofluorescence microscopy

Grain sections embedded in LR White resin were pre-incubated (50 µL drop per well) in 3 % (w/v) bovine serum albumin (BSA; Fraction V, A 2153, Sigma) in phosphate-buffered saline at pH 7·4 for 30 min, then incubated in phosphate-buffered saline with 1 % (w/v) bovine serum albumin (BSA) for 2 h in primary antibody. The following monoclonal and polyclonal antibodies were used, singly or in combination, diluted in phosphate-buffered saline containing 1 % (w/v) BSA and 0·05 % (w/v) Tween-20: rabbit polyclonal anti-prolamin diluted 1:400, rabbit polyclonal anti-glutelin diluted 1:400 (Krishnan and Okita, 1986), mouse monoclonal anti-IFRN 1602 (Mills *et al.*, 2000) diluted 1:10, and rabbit polyclonal anti-HMW-R2 (Denery-Papini *et al.*, 1996) diluted 1:100. Slides were rinsed three times for 5 min with phosphate-buffered saline–Tween, then incubated for 2 h, in the dark, with secondary antibody (anti-rabbit Alexa 488 conjugated and/or anti-mouse Alexa 568 conjugated, Invitrogen) diluted 1:200 in phosphate-buffered saline, 1 % (w/v) BSA, 0·5 % (w/v) Tween. Slides were then rinsed twice with phosphate-buffered saline–Tween, twice with phosphate-buffered saline and three times with water. Samples were analysed with a Zeiss 780 laser scanning microscope.

### Immuno-transmission electron microscopy (TEM)

A similar protocol to that described above was also used for immuno-TEM studies of ultrathin sections from the same samples. In this case, grids were floated on 50 µL drops and the secondary antibody (goat anti-rabbit conjugated to 15 nm gold) (British BioCell International) was used at a 1:50 dilution.

Gold-labelled sections were silver enhanced using an R-Gent SE-EM Silver Enhancement Reagents kit from AURION according to the manufacturer's protocol.

## RESULTS

The production and characterization of transgenic rice lines (*O. sativa* ‘Dongjin’) expressing the chromosome 1D-encoded (*Glu Dx5*) HMW-GS 1Dx5 under the control of its own starchy endosperm-specific promoter have been previously described (Oszvald *et al.*, 2007). The expression level of the HMW-GS encoded by the transgene was determined by densitometric analysis of polyacrylamide gels (Oszvald *et al.*, 2007). Line 26 showed the highest expression level for the transgenic protein, which represented about 3·81 % of the total alcohol-soluble proteins, and was therefore selected for the present study.

The specificities of the antibodies raised against rice storage proteins (prolamin and glutelin) were determined by western blot analysis (Supplementary Data Fig. S1). This showed that neither antibody reacted with proteins in extracts of wheat whole grain (‘Cadenza’), but that the anti-prolamin antibody showed some binding to the major rice glutelin component. In contrast, no binding of the anti-glutelin antibody to rice prolamins was observed. The specificity of the IFRN 1602 and anti-R2-HMW antibodies raised against the HMW-GS epitopes was also tested against wheat and rice endosperm proteins. Both antibodies reacted specifically with HMW-GS extracted from wheat whole grain but not with other wheat gluten proteins or with rice prolamin or glutelin fractions. Both antibodies recognized the transgenic HMW-GS expressed in rice endosperm (Supplementary Data Fig. S1).

A detailed analysis of the distribution of HMW-GS 1Dx5 in the transgenic rice endosperm was made by light microscopy using immunological staining.

### Patterns of expression of the wheat protein sub-unit

Localization of the transgenic HMW-GS, at both the cellular and sub-cellular levels, was carried out by immunofluorescence microscopy using the HMW-GS-specific antibodies IFRN 1602 and anti-R2-HMW.

Double labelling using the monoclonal IFRN 1602 antibody in combination with the polyclonal antibodies recognizing native rice prolamin or glutelin proteins was used to determine in which type of protein body (ER or vacuolar) the transgenic HMW-GS was deposited. In these double labelling experiments, the primary antibodies were detected using secondary antibodies conjugated to two different fluorochromes, AlexaFluor-conjugated 488 and 568, which do not overlap in their excitation or emission spectra. The polyclonal anti-R2-HMW sub-unit antibody could not be used for double labelling with the anti-rice prolamin and glutelin antibodies as these antibodies were all raised in rabbits, meaning that it was not possible to use specific secondary antibodies. Therefore, the IFRN 1602 antibody was selected for double labelling studies. Double labelling of developing seeds was carried out at 7, 12, 14, 17, 20 and 25 daf.

**Fig. 1.**
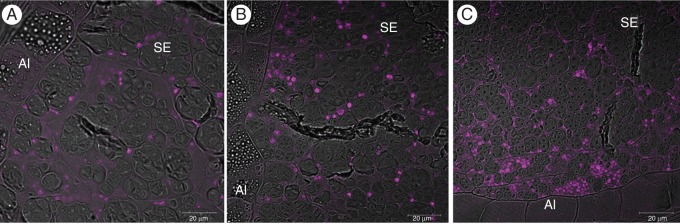
Confocal microscope images of transgenic rice endosperm showing the immunofluorescence labelling (magenta fluorescence) pattern obtained with the IFRN 1602 primary antibody, which specifically recognize HMW glutenin sub-units, detected with an anti-mouse secondary antibody conjugated to Alexa 568. (A) 7 daf; (B) 12 daf; (C) 17 daf. SE, starchy endosperm; Al, aleurone layer.

Figure 1 shows immunofluorescence labelling of endosperm sections with the IFRN 1602 antibody. Specific labelling of the protein bodies in the starchy endosperm (Fig. 1A–C), but not of those in the aleurone cells, was observed. In the early stages of development, the protein bodies labelled by IFRN 1602 appeared to be evenly distributed in cells across the starchy endosperm (Fig. 1A, B), while at later stages of development (Fig. 1C) the labelling appeared to be more dense in the sub-aleurone cells. A similar pattern of labelling was observed when the anti-R2-HMW antibody was used (data not shown).

**Fig. 2.**
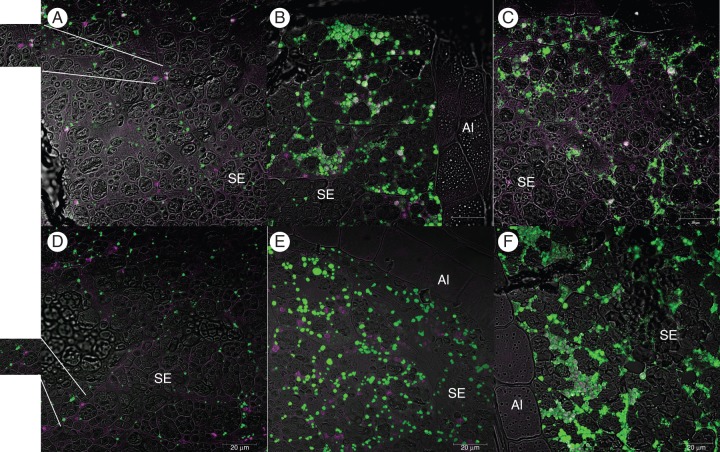
Double immunofluorescence labelling of rice grain sections at 7 (A, D), 14 (B, E) and 20 (C, F) daf showing the locations of the transgenic HMW glutenin sub-unit and endogenous prolamins and glutelins in the endosperm. The anti-mouse secondary antibody used for the detection of IFRN 1602 was conjugated to Alexa 568 (magenta), while the anti-rabbit secondary antibody used to detect either the rice anti-prolamin antibody (A, B, C), or the anti-glutelin antibody (D, E, F), was conjugated to Alexa 488 (green). SE, starchy endosperm; Al, aleurone layer.

Double labelling using the IFRN 1602 antibody in combination with the rice prolamin or glutelin antibodies is shown in Fig. 2. Protein bodies labelled in magenta contain the HMW-GS while those labelled in green contain prolamin (Fig. 2A–C) or glutelin (Fig. 2D–F). Protein bodies stained light grey (resulting from the combination of the green and magenta colours) show co-deposition of the transgenic HMW-GS with the endogenous rice storage proteins.

In the 7 daf sections, the wheat HMW-GS was observed both in PB-Is (so identified because they were stained by the prolamin antibody; Fig. 2A) and in separate protein bodies that did not contain rice prolamins (see inset in Fig. 2A). Likewise, not all PB-Is contained the transgenic sub-unit.

In contrast, double labelling for the HMW-GS and rice glutelin (Fig. 2D) showed that the two proteins were largely segregated into different protein bodies in the 7 daf endosperm.

The extent of co-localization of the HMW-GS with the rice prolamin appeared to increase as the grain matured (Fig. 2B, C), since a higher proportion of protein bodies is stained in shades of grey in grains at 14 and 20 daf than in grains at 7 daf, although protein bodies labelled only by the green fluorochrome, and therefore containing only prolamins, are still visible in the more mature stages. Co-localization of the transgenic protein with the rice glutelins was not observed by confocal microscopy with a ×25 magnification objective in young grains (up to 14 daf) (Fig. 2E), indicating that none or only a very a limited amount of the recombinant protein is transported to the storage vacuole in the early stages of grain development. Co-localization of the HMW-GS with the endogenous rice glutelins appeared obvious, however, in 20 daf grains (Fig. 2F).

None of the antibodies was labelled in the extracellular space, demonstrating that the proteins were not secreted from the cells.

### Transmission electron microscopy observations of wild-type and transgenic grains, and localization of the HMW-GS in endosperm cells

The intracellular deposition of the endogenous rice endosperm proteins and of the wheat HMW-GS was studied by immunoelectron microscopy. Ultrathin sections corresponding to regions of the endosperm two to three cell layers from the aleurone were prepared from developing seeds at 7, 12, 14, 17, 20 and 25 daf and were then probed with either the anti-glutelin, the anti-prolamin or the anti-R2-HMW polyclonal antibody or combinations of these. The primary antibodies were detected with a secondary antibody conjugated to 10 nm gold particles.

**Fig. 3.**
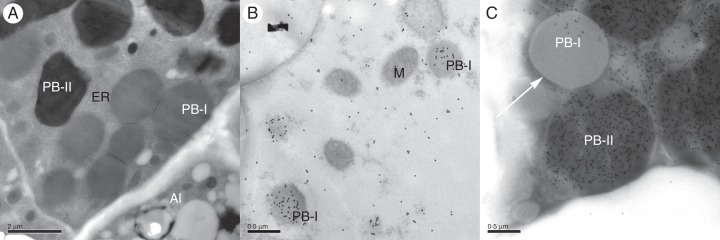
Transmission electron micrograph of sub-aleurone cells of the starchy endosperm of wild-type rice at different development stages. (A) 14 daf rice; (B) 7 daf rice labelled with rice anti-prolamin antibody detected with gold-conjugated secondary antibody (10 nm gold particles); (C) 17 daf grain labelled with rice anti-glutelin antibody (10 nm gold particles). ER, endoplasmic reticulum; PB-I, type I protein body; PB-II, type II protein body; Al, aleurone layer; M, mitochondrion. The white arrow indicates the typical PB-I of rice.

**Fig. 4.**
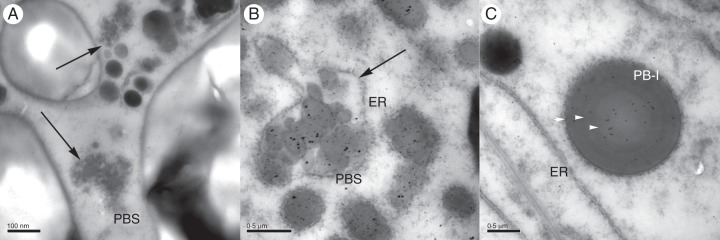
Details of sections of starchy endosperm from transgenic rice grains at different developmental stages. (A) 7 daf; (B and C) 14 daf. (A) A novel type of protein body (PBS), indicated by arrows, is visible in transgenic rice endosperm. (B) 14 daf: double labelling with anti-R2-HMW and rice prolamin antibodies detected with gold-conjugated secondary antibody. The novel type of protein bodies (PBSs) are labelled by both antibodies. The small (10 nm) gold particles identify the prolamin proteins; the larger (silver-enhanced) particles identify the HMW 1Dx5 glutenin sub-unit. (C) 14 daf: PB-I labelling by the anti-R2-HMW antibody (10 nm gold particles). ER, endoplasmic reticulum; PB-I, type I protein body; PB-II, type II protein body. Black arrows indicate the novel type of protein bodies (PBSs); white arrowheads indicate the concentric ‘rings’ on PB-I.

The sections showed good preservation of the ER and other cell organelles (Figs 3 and 4). The two types of protein bodies (PB-I and PB-II) (Krishnan and White, 1995), which are typical of rice endosperm, are clearly distinguishable in the non-transgenic wild-type rice plants (Fig. 3). PB-Is are smooth, spherical and heavily labelled by the antibody specific for rice prolamins (Fig. 3B). They have diameters of about 0·3–0·6 µm in the early stages of development (7 daf) (Fig. 3B) which increase to about 1–1·5 µm in the 17 daf grain, and the larger protein bodies have a lamellar structure consisting of concentric rings (Fig. 3C, arrows; Fig. 4C, arrowheads).

In contrast, PB-IIs are irregularly shaped (Fig. 3A, C), generally larger than PB-Is (up to 3–4 µm diameter in 17 daf grains), and strongly labelled by the anti-glutelin antibody (Fig. 3C).

### Appearance of a new type of protein body in transgenic rice endosperm

Analyses of developing grain of transgenic rice expressing the wheat HMW-GS showed the presence of PB-Is and PB-IIs with similar sizes and staining properties to those in the wild-type grain. However, a third type of protein body was also observed (Fig. 4A, B, arrows). These resemble PB-Is in being spherical, but are much smaller (0·1–0·2 µm diameter), lack the lamellar structure of typical PB-Is and form clusters surrounded by a ribosome-studded membrane which we suggest may represent dilated ER (Fig. 4A, B, arrows).

Such protein body structures (PBSs) were observed only in the endosperm cells of transgenic seeds, in both early and later stages of development (Fig. 4A, B), although they appeared to be more abundant in the younger grains: the ratio of PBSs to normal PB-Is was about 1:3 in 7 and 14 daf grains, whereas in 17 and 25 daf grains the ratio was closer to 1:5. Double labelling experiments were carried out using anti-R2-HMW in combination with either rice glutelin or rice prolamin antibodies. A 10 nm gold-conjugated anti-rabbit secondary antibody was used against the anti-R2-HMW antibody, and the size of the gold particles was subsequently increased by silver enhancement. The sections were then subjected to a second labelling step using antibodies specific for prolamin or glutenin detected with anti-rabbit secondary antibody conjugated to 10 nm gold particles.

**Fig. 5.**
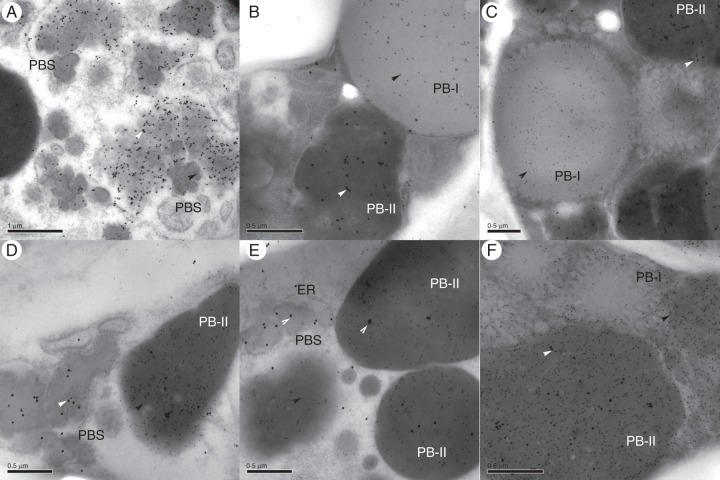
Double labelling of ultrathin sections from transgenic rice samples in different developmental stages. (A and D) 14 daf; (B and E) 17 daf; (C and F) 25 daf. (A–C) Double labelling with anti-R2-HMW and rice prolamin antibodies. (D–F) Double labelling with anti-R2-HMW and rice glutelin antibodies. The small (10 nm) gold particles identify the prolamin/glutelin proteins (black arrowheads); the larger (silver enhanced) gold particles identify the HMW Dx5 GS proteins (white arrowheads). PBS, a novel type of protein body.

In early stages of development (7–14 daf) PBSs in the transgenic rice reacted strongly with the antibody specific for the wheat HMW-GS (anti-R2-HMW) and with antibody for rice prolamins (Figs 4B and 5A); in these early stages, the normal PB-Is containing rice prolamins were often also labelled by the anti-R2-HMW antibody (Fig. 4C) while only a very low level of labelling of the PB-IIs with the anti-R2-HMW antibody was observed (Fig. 5D) and only in 14 daf grains. These results indicate that the wheat HMW-GS is initially deposited in the same protein body as the rice prolamins, suggesting a common origin for PB-Is and the novel type of protein body (PBS).

Labelling with the anti-R2-HMW antibody appears more dense in PB-IIs in 17 daf and 25 daf grains than in younger grains (compare Fig. 5B and C with D). PB-Is devoid of any labelling for the transgenic sub-units were observed in 17 and 25 daf sections (Fig. 5B, C), similarly to what had been already observed in double immunofluorescent labelling experiments with the prolamin and the IFRN 1602 antibodies. In 25 daf transgenic rice (Fig. 5C, F), unlike in wild-type rice (see Supplementary Data Fig. S2), the integrity of the protein bodies appeared to be disrupted in some regions of the endosperm (mainly in the inner layers), suggesting that a process of protein matrix formation similar to that observed in wheat endosperm (Parker, 1980; Tosi *et al.*, 2009; Tosi, 2012) may be occurring.

### The wheat HMW-GS forms alcohol-insoluble polymers in rice endosperm

**Fig. 6.**
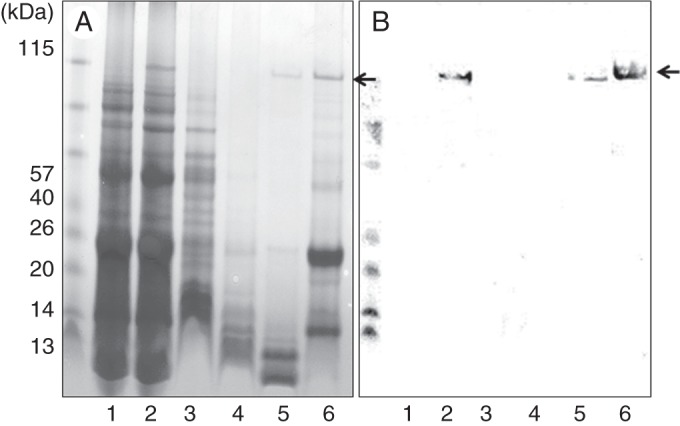
Analysis of storage proteins in wild-type and transgenic rice endosperm. (A) SDS–PAGE and (B) western blot analysis with the anti-R2-HMW antibody of protein fractions extracted from whole grains of transgenic rice lines. 1, Total protein extract from the wild-type rice line; 2, total protein extract from a transgenic rice line (matured seed); 3–6, protein fractions extracted from transgenic rice seed; 3, albumin/globulin proteins fraction extracted with 0·5 M NaCl; 4, cysteine-poor prolamin proteins extracted with 60 % *n*-propanol without DDT; 5, cysteine-rich fraction prolamin proteins extracted with 60 % *n*-propanol with 1 % (w/v) DDT; 6, protein extracted from the residue using protein extraction buffer with DDT. Arrows indicate the HMW Dx5 GS protein.

Sequential extraction of grain proteins and separation by SDS–PAGE was carried out to determine the polymerization state of the recombinant wheat HMW-GS in the mature rice grain. Milled grain was initially treated with 0·5 m NaCl to extract albumin and globulin proteins followed by 60 % (v/v) propan-1-ol to extract rice prolamins, and 60 % (v/v) propan-1-ol with 1 % (w/v) DTT to extract rice prolamins present as insoluble polymers. This was followed by extraction with buffer containing urea to extract rice glutelins and other insoluble proteins. The wheat HMW-GS was only observed in the prolamin fraction extracted by 60 % propan-1-ol with DTT and in the glutelin fraction (Fig. 6A), and only from the transgenic seeds, its identity being confirmed by western blot analysis with anti-R2-HMW antibody (Fig. 6B).

The recombinant HMW-GS therefore forms insoluble deposits with similar properties to the polymeric rice prolamins and insoluble rice glutelins. This could result from molecular interactions between the HMW-GS and rice proteins, or from the HMW-GS forming insoluble polymers as in wheat.

## DISCUSSION

Cereal grain storage proteins are typical secretory proteins in that their synthesis, folding and deposition take place within the endomembrane system of the plant cell (Bollini and Chrispeels, 1979). Both rice and wheat storage protein are therefore synthesized on polyribosomes attached to the ER and contain N-terminal signal peptide sequences that mediate their passage into the lumen of the ER. In rice, the two main type of storage proteins, prolamin and glutelin, are synthesized in different regions of the ER and packaged into separate types of protein body, called PB-Is and PB-IIs, respectively. Whereas the prolamins remain in the ER lumen, which then develops into PB-Is, the glutelin precursor is transported first from the ER lumen to the Golgi and then to a protein storage vacuole (PSVs), which eventually form PB-IIs. These two population of protein bodies remain distinct throughout development and in the mature grain. It is now widely accepted that two trafficking pathways also occur in wheat endosperm, with the major storage proteins, the gluten proteins, either being transported via the Golgi apparatus into the vacuole or accumulating directly within the lumen of the ER. However, storage protein deposition and protein body formation in the wheat endosperm presents some important differences when compared with rice. In particular, the basis for the sorting of the different types of gluten protein within the two pathways remains unclear, but both the intrinsic properties of the protein and the age of the grain may play a role. Segregation of gluten proteins both between and within protein bodies has been observed (Shewry, 1999; Tosi, 2012), particularly in grain at early to mid-development, while the two population of protein bodies appear largely to merge in mature seeds, and it has been suggested that the protein bodies of ER origin are transported to the PSV via a Golgi-independent mechanism resembling autophagy (Levanony *et al.*, 1992). Furthermore, the same individual proteins have been reported to follow either of the trafficking pathways, depending on the stage of development of the endosperm tissue (Tosi *et al.*, 2009).

Seeds are an attractive system for the production of recombinant proteins (Stoger *et al.*, 2005; Takaiwa *et al.*, 2007), with the ER, ER-derived protein bodies, PSVs, apoplast and cytosol being alternative sites for the deposition and accumulation of recombinant proteins in the rice endosperm. However, recombinant proteins do not always follow the patterns of deposition in transgenic cereal endosperm that would be expected on the basis of their targeting signals. Arcalis *et al.* (2004) expressed a recombinant human serum albumin tagged with an ER retrieval signal (KDEL), a fungal phytase designed for secretion and a recombinant legumin containing structural information for targeting to the vacuole in transgenic wheat endosperm and found that all three recombinant proteins were deposited in the vacuole. The authors suggested that the unexpected patterns of trafficking and deposition of the recombinant proteins they observed could be related to the specialized architecture of endosperm cells, which are designed for storage.

We have expressed the wheat HMW-GS 1Dx5 under the control of its own promoter in transgenic rice endosperm and studied its deposition in relation to the endogenous rice storage proteins, at both the tissue and intracellular levels, using a combination of immunofluorescence confocal laser scanning microscopy and immuno-TEM.

Whereas the HMW glutelin sub-units are concentrated in the inner part of the starchy endosperm in wheat (Tosi *et al.*, 2009), the transgenic rice showed more intense immunolabelling in the protein bodies of the sub-aleurone layer when a HMW-GS-specific antibody was used for detection. Since the recombinant HMW-GS was expressed under the control of its own promoter, these results indicate that the same promoter conferred subtly different patterns of expression in the starchy endosperm tissues of the two cereals. This could result from differences in the timing of endosperm differentiation (and in particular formation of the sub-aleurone layer) and expression of the transgenic HMW-GS in wheat and rice, or reflect a different distribution in the two cereals of specific regulatory signals.

Double immunofluorescence labelling was also carried out to determine the deposition of the wheat glutenin sub-unit relative to that of the rice glutelin and prolamin storage proteins in the same tissues and cells. Co-localization of the recombinant wheat HMW-GS with both main rice storage proteins was observed. In earlier stages of development, labelling specific for the transgenic protein was observed on PB-Is and, more abundantly, on a new type of protein bodies, which we named PBSs, and that were present only in the transgenic plants. In contrast, at later stages of development, the recombinant sub-unit was detected mainly in PB-IIs. The presence of a membrane with associated ribosomes surrounding clusters of these new type of protein bodies implies that, similarly to PB-Is, they originated from deposition of protein in the ER. The heavy labelling of the PBSs by the rice prolamin antibody suggests, in fact, that they may represent PB-Is whose normal development has been affected by the co-deposition of the HMW glutenin sub-unit with the endogenous rice prolamins. Typical PB-Is containing endogenous prolamins but no transgenic HMW glutenin were also observed (green-stained protein bodies in Fig. 2A and B), indicating that a certain level of segregation in ER sub-domains exists between the transgenic sub-unit and the endogenous prolamins. This may simply reflects the fact that compartmentalization may exist within different rice prolamins, but could not be detected given the broad spectrum nature of the prolamin antibody.

Abnormal or new types of protein bodies have also been reported in mutant rice lines accumulating high amounts of the 57 kDa glutelin precursor and correspondingly low amounts of the glutelin acidic and basic sub-units (Kumamaru *et al.*, 1988; Takemoto *et al.*, 2002) and in transgenic rice lines expressing other recombinant proteins (Yang *et al.*, 2003, 2007; Saito *et al.*, 2009; Shigemitsu *et al.*, 2013), and it has been suggested that they may result from perturbations in the intracellular trafficking and sorting of native storage proteins (Yang *et al.*, 2003), due either to the high levels of expression of the recombinant proteins or to interactions of the recombinant proteins with native storage proteins or with co-expressed recombinant proteins (Takaiwa *et al.*, 2008).

The fact that the recombinant HMW-GS is only extracted under reducing conditions indicates that it is able to form disulfide-stabilized polymers in the grain. However, it remains unclear whether the recombinant sub-unit forms disulfide bonds with endogenous prolamins and/or glutelins, or only with other recombinant sub-units. The formation of disulfide bonds with endogenous proteins could be responsible for the deposition in PB-Is and PB-IIs: the recombinant protein may be co-transported and follow the trafficking pathway of the endogenous proteins.

Several studies have underlined the essential role played in the formation of ER-derived PB-Is in rice by the interaction of prolamins with the ER chaperone BiP (Li *et al.*, 1993; Muench *et al.*, 1997; Saito *et al.*, 2009). BiP is always highly enriched on the periphery of PB-Is (Muench *et al.*, 1997), suggesting its involvement not only in the ER retention of rice prolamins, but also in their deposition and therefore in the structural organization of these protein bodies.

In wheat, in contrast, the role of BiP in the retention of prolamins within the ER and its contribution to the formation of ER-derived protein bodies remain unclear. The irregularly shaped protein bodies observed in our study may therefore result from disruption of the ordered deposition of the endogenous prolamins, possibly because the interactions between the recombinant protein and endogenous prolamins prevented the proper interaction of the latter with BiP.

Disturbance of the structural organization of the protein bodies could also make them less stable, resulting in the partial loss of integrity of protein bodies observed in the mature endosperm of transgenic rice (Fig. 5F). This could lead to the merging of the PBS with the vacuolar protein bodies (PB-IIs), accounting for the increased presence of the HMW-GS in PB-IIs in older grains.

An alternative explanation for the patterns of trafficking and deposition observed for endogenous and recombinant HMW glutenin sub-units in transgenic rice could be that the expression of the recombinant protein results in a more generic malfunctioning of the ER sorting machinery. Proteomic analysis of the transgenic line (Oszvald *et al.*, 2013) showed that the levels of some molecular chaperones were significantly higher compared with non-transformed, control rice lines. This suggests that although the recombinant HMW-GS represents <0·8 % of the total storage proteins, its expression may be enough to trigger the ER stress response and therefore affect protein sorting and PB formation.

A unique feature of plants is that they are able to store proteins in the ER in addition to other endomembrane compartments, and the deposition of such storage proteins provides important sources for both human and animal nutrition. Thus, increasing our knowledge of the mechanisms governing the targeting of storage proteins to the different organelles will ultimately allow the modification of critical steps in this process, leading to improvements of crop plants as a protein source.

Our study contributes to our understanding of storage protein trafficking and deposition in rice and wheat seeds, suggesting that while the intrinsic properties of recombinant proteins appear to determine their initial sites of accumulation, their final pattern of deposition within the cell may be determined by which other proteins are expressed in that same cell, their intrinsic properties and their interaction with the recombinant protein.

## SUPPLEMENTARY DATA

Supplementary data are available online at www.aob.oxfordjournals.org and consist of the following. Figure S1: western blot analysis of rice and wheat grain protein extracts with rice glutelin, rice prolamin, R2-HMW and IFRN 1602 antibodies. Figure S2: ultrathin section from wild-type rice samples (25 daf).

Supplementary Data
