# Supplementary material for: The trafficking pathway of a wheat storage protein in transgenic rice endosperm
Source: Ann Bot. 2014 Mar 5;113(5):807–15. doi: 10.1093/aob/mcu008 (PMC3962248; doi:10.1093/aob/mcu008)
Supplement: Supplementary Data [file supp_113_5_807__index.html]

The trafficking pathway of a wheat storage protein in transgenic rice endosperm — The trafficking pathway of a wheat storage protein in transgenic rice endosperm — Supplementary Data 

# The trafficking pathway of a wheat storage protein in transgenic rice endosperm

## Supplementary Data

Supplementary Data

**Files in this Data Supplement:**

- Supplementary Data - Pdf file
