# Supplementary material for: The trafficking pathway of a wheat storage protein in transgenic rice endosperm
Source: Ann Bot. 2014 Mar 5;113(5):807–15. doi: 10.1093/aob/mcu008 (PMC3962248; doi:10.1093/aob/mcu008)

SUPPLEMENTARY DATA

Fig. S1. Western blot analysis of rice and wheat grain protein extracts with rice glutelin (A), rice prolamin (B), R2- HMW (C) and IFRN 1602 (D) antibodies. (A, B) 1: total protein extract from wheat ('Cadenza'); 2–4: transgenic rice lines. (C) 1: total protein extract from whole grain of wheat; 2: wild type rice; 3–5: transgenic rice lines. (D) 1: wild type rice; 2: transgenic rice line; 3: wheat proteins.

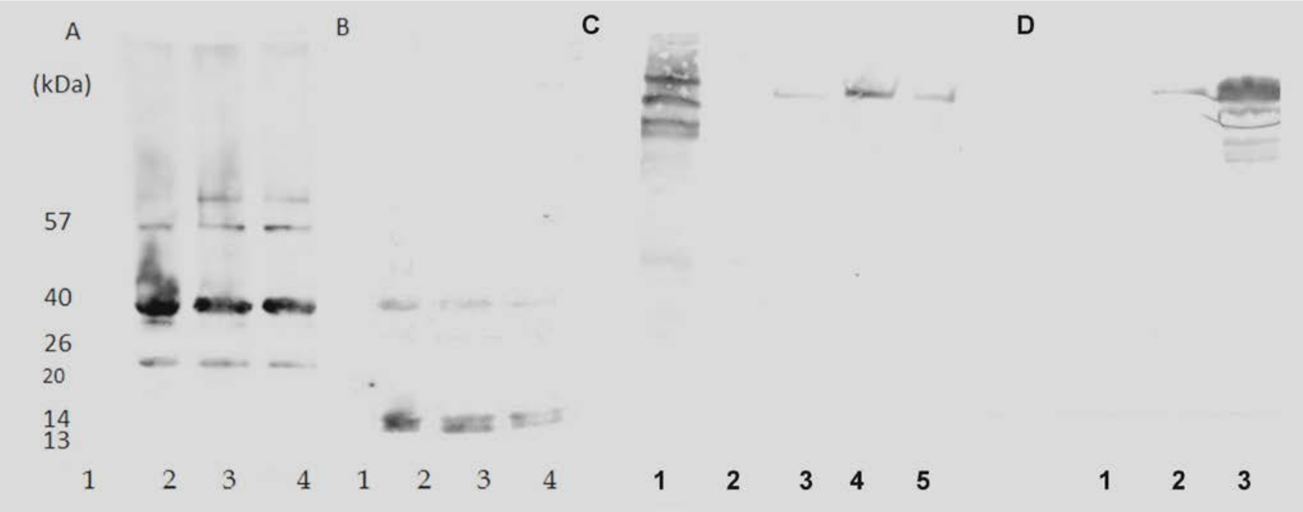

Fig. S2. Ultrathin section from wild type rice samples (25 daf). PB-I = protein body containing prolamin; BP-II = protein body containing glutelin.

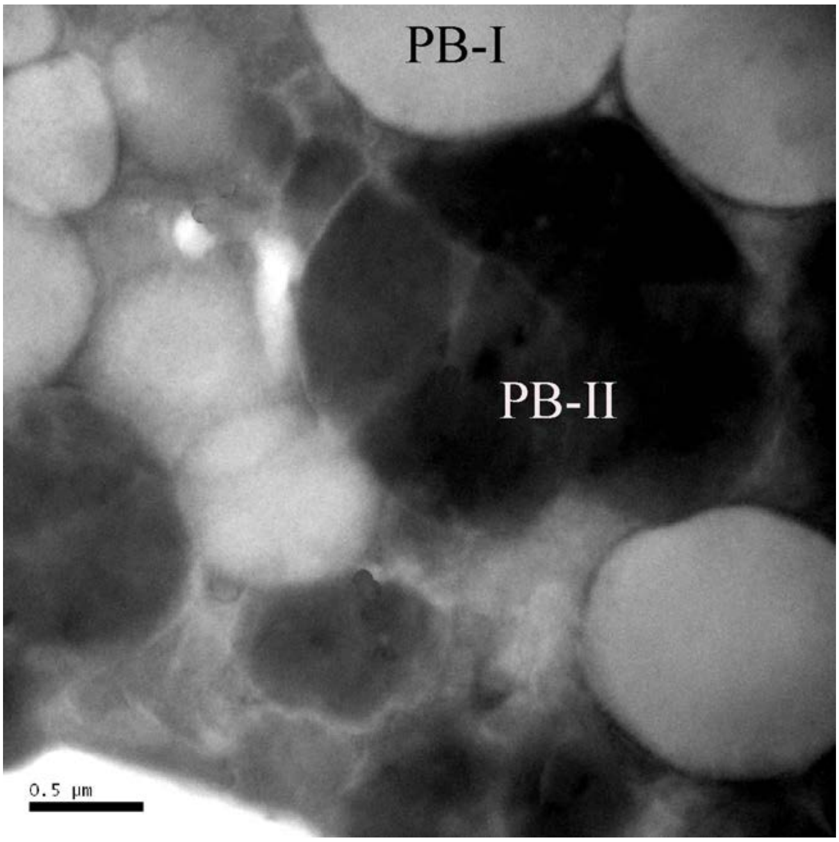

Supplement: Supplementary Data [file supp_mcu008_mcu008supp.pdf]
